# Staggered Onsets of Processing Relevant and Irrelevant Stimulus Features Produce Different Dynamics of Congruency Effects

**DOI:** 10.5334/joc.252

**Published:** 2023-01-13

**Authors:** Herbert Heuer, Christian Seegelke, Peter Wühr

**Affiliations:** 1 Leibniz Research Center for Working Environment and Human Factors (IfADo), DE; 2 Department of Psychology, University of Salzburg, Salzburg, Austria; 3 Centre for Cognitive Neuroscience, University of Salzburg, Salzburg, Austria; 4 TU Dortmund University, DE

**Keywords:** Action and perception, Mathematical modeling, Response speed

## Abstract

The dynamics of congruency effects in conflict tasks can be analyzed by means of delta plots which depict the reaction-time differences between incongruent and congruent conditions across the quantiles of the reaction-time distributions. Delta plots exhibit a variety of different shapes. Here we test the hypothesis that staggered onsets of processing task-relevant and task-irrelevant features for response selection (together with a declining influence of the irrelevant feature) produce such variety. For this purpose, staggered onsets were implemented in two extensions of the Leaky, Competing Accumulator model. We show the cardinal capability of these models to produce different shapes of delta plots with different assumptions about temporal offsets between processing relevant and irrelevant stimulus features. Applying the models to experimental data, we first show that they can reproduce the delta plots observed with a conflict task with stimulus size as the irrelevant feature. For this task congruency effects are delayed and appear only at longer reaction times. Second, we fit the models to the results of two new Simon-task experiments with an experimentally controlled temporal offset in addition to the internal one. The experimentally induced variations of the shape of delta plots for this task could be reasonably well fitted by one of the two models that assumed an early start of response selection as soon as either the relevant or the irrelevant stimulus feature becomes available. We conclude that delta plots are crucially shaped by staggered onsets of processing relevant and irrelevant features for response selection.

## Introduction

In conflict tasks, participants respond intentionally to a task-relevant feature of a stimulus, but in addition, there is an unintended influence of a task-irrelevant feature on reaction time and accuracy. A prominent example is the Simon task (Lu & Proctor, 1995; Simon, 1969). In this task, a typical relevant feature is the color of a circle, and the irrelevant feature is the position of the circle on the left or right side of a fixation point. When responses to two different colors are made with the left or right hand, the irrelevant feature dimension (left vs right position of the stimulus) overlaps with the spatial response dimension (left vs right position of the response key). With this dimensional overlap, the irrelevant stimulus feature can be congruent or incongruent with the relevant response feature and thereby facilitate or inhibit responding (cf. Kornblum, Hasbroucq, & Osman, 1990).

The difference between congruent and incongruent conditions of a conflict task, the congruency effect, can be observed in mean reaction times and accuracies. However, the means of the reaction-time distributions provide no information on the dynamics of the congruency effect, that is, on its time course across the range of reaction times. Such information is provided by so-called effect functions (e.g., Wiegand & Wascher, 2005) or delta plots (e.g., Van den Wildenberg, Wylie, Forstmann, Burle, Hasbroucq, & Ridderinkhof, 2010) which show the congruency effect across the bins of vincentized reaction-time distributions (cf. Ratcliff, 1979). Here we give a brief overview of the variety of delta plots reported in the literature and propose a specific hypothesis to account for a major part of the diversity, namely staggered onsets of processing task-relevant and task-irrelevant features. We implement this hypothesis in two extensions of sequential-sampling models for conflict tasks and test it with four data sets, two taken from the literature and two new ones.

## Dynamics of congruency effects

Mean reaction times in congruent and incongruent conditions of conflict tasks fall short of exploiting the information inherent to the reaction-time distributions. This is amended by the analysis of congruency effects for vincentized distributions (or for selected quantiles of the distributions). In vincentizing a distribution, mean reaction times are computed for bins bounded by increasing quantiles. For example, quantiles can be defined by 20% steps, so that there are five bins. The results of such analyses are often presented in the format of delta plots where the sample means of the differences between the individual means in incongruent and congruent conditions are plotted against the sample means of the averages of these individual means for each of the bins of the reaction-time distributions. This type of analysis has been introduced by De Jong, Liang, and Lauber (1994). The results are considered to reflect the time course of congruency effects and thus the time course of the activation by irrelevant stimulus features (cf. Proctor, Miles, & Baroni, 2011).

Delta plots are shaped by the differences between reaction-time distributions in incongruent and congruent conditions, and vice versa, they allow certain inferences about the relation between these distributions (Speckman, Rouder, Morey, & Pratte, 2008). For example, the variability of reaction times typically increases with the mean (Wagenmakers & Brown, 2007), and often not only mean reaction time is longer in the incongruent condition of a conflict task than in the congruent condition, but reaction-time variability is larger as well. A by-product of this difference is an increasing delta plot (Zhang & Kornblum, 1997). In fact, the most common type of delta plot is a monotonic increase with increasing reaction time, with congruency effects already present for the fastest responses. Such increase is typical for the Eriksen flanker task (Burle, Spieser, Servant, & Hasbroucq, 2014; Eriksen & Eriksen, 1974, Mittelstädt, Miller, Leuthold, Mackenzie, & Ulrich, 1921) or the Stroop task (Pratte, Rouder, Morey, & Feng, 2010; Stroop, 1935). However, the classical Simon task typically exhibits decreasing delta plots (Burle, Possamaï, Vidal, Bonnet, & Hasbroucq, 2002; De Jong et al., 1994; Mittelstädt et al., 2021; Wiegand & Wascher, 2007), though with vertically arranged positions of stimuli and responses delta plots tend to increase (cf. Töbel, Hübner, & Stürmer, 2014; Vallesi, Mapelli, Schiff, Amodio, & Umiltà, 2005; Wiegand & Wascher, 2005; for an exception see Wühr & Biebl, 2011). Decreasing delta plots have also been observed for Eriksen flanker task when arrows or orientations of gratings served as stimuli (Pratte, 2021).

Reviewing a large range of Simon-task variants, Proctor et al. (2011) listed the following types of delta plots: decreasing, increasing, stable, stable then decreasing, stable then increasing, increasing then decreasing. Essentially all the observed delta plots – decreasing as well as increasing ones – start with a congruency effect already at the fastest bin of the reaction-time distributions. Contrary to this typical feature, in a re-analysis of data of Wühr and Seegelke (2018, Exp. 2) and Richter and Wühr (2022, Exp. 2), who studied a conflict task with color as the task-relevant stimulus feature and stimulus size as the task-irrelevant one, we observed delta plots that were initially close to zero, and congruency effects emerged only at longer reaction times. Similar delta plots with delayed appearance of congruency effects have been observed before in a variant of the Simon task when irrelevant spatial stimuli were presented with short delays after presentation of the relevant color stimuli (Burle, van den Wildenberg, & Ridderinkhoff, 2005). This similarity of findings suggests a role of a delayed onset of processing the irrelevant stimulus feature for the particular shape of delta plots with delayed congruency effects even when temporal offsets are not experimentally controlled. These findings motivated the current extensions of sequential-sampling models.

## Theoretical implications of different shapes of delta plots

Given the typical increase of the variability of reaction times with their mean (Wagenmakers & Brown, 2007), increasing delta plots appear more or less natural and do not call for explanations related to the nature of particular tasks. In contrast, decreasing delta plots violate the normal relation between means and variances of reaction times and call for a dedicated explanation. The currently prevailing account assumes initial activation by the irrelevant stimulus feature, which produces the congruency effect for the shortest reaction times, followed by subsequent suppression (e.g., Ridderinkhof, 2002; Van den Wildenberg et al., 2010) or decay (Hommel, 1994), which produces the decline of the congruency effect and – in case suppression is too strong – even its reversal (e.g., Hübner & Mishra, 2013). The activation-suppression hypothesis can be conceived as an extension of the dual-route model (Kornblum et al., 1990): the notion that relevant and irrelevant stimulus features are processed via distinct, but converging routes, is supplemented with the hypothesis of different dynamics by positing that the initial activation along the automatic route – and thus the activation by the irrelevant stimulus feature – is suppressed later on (cf. Kornblum, Stevens, Whipple, & Requin, 1999; Simon, Acosta, Mewaldt, & Speidel, 1976; Zorzi & Umiltá, 1995).

The activation-suppression account of decreasing delta plots faces a somewhat subtle problem that is rooted in the fact that reaction times measure durations of processes, but not current states (cf., Heuer, 1981; McLeod, 1980). The analogy of a race illustrates the problem: immediately after the start, runner A (representing a congruent condition) is faster than runner B (representing an incongruent condition) as a result of the initial activation and inhibition, respectively, by the irrelevant stimulus feature. When the initial effect of the irrelevant stimulus feature is suppressed later, runner A will lose its advantage in terms of speed, but will nevertheless reach the finishing line earlier than runner B. Thus, the early activation/inhibition by congruent and incongruent task-irrelevant stimulus features will produce a reaction-time difference that will persist even when the effect of the irrelevant stimulus feature is fully suppressed later on. The reaction-time difference will be reduced only when the suppression of the instantaneous effect of the irrelevant feature is turned into its reversal so that an activating effect becomes inhibiting and vice versa. Suppression of the instantaneous influence of task-irrelevant stimulus features by itself cannot account for decreasing delta plots.

The subtle problem of the activation-suppression account of decreasing delta plots becomes less subtle when one considers formal models of speeded decisions in conflict tasks. Sequential-sampling models are the dominant processing models for speeded decisions and thus for reaction-time tasks (Evans & Wagenmakers, 2020; Forstmann, Ratcliff, & Wagenmakers, 2016). Their basic assumption is the progressive accumulation of evidence (or activation) until a criterion is reached at which a response is initiated (cf. Bogacz, Brown, Moehlis, Holmes, & Cohen, 2006; Ratcliff, Smith, Brown, & McKoon, 2016; Smith & Ratcliff, 2004). Until about a decade ago, decreasing delta plots were considered to be at odds with this class of models (cf. Pratte et al., 2010).

In more recent years, sequential-sampling models with suppression or passive decay of the activation by a task-irrelevant stimulus feature have been proposed for the Eriksen flanker task (e.g., Hübner, Steinhauser, & Lehle, 2010; White, Ratcliff, & Starns, 2011). For this task, delta plots typically do not decline. Decreasing delta plots, as observed for the Simon task, will emerge when the instantaneous effect of the irrelevant stimulus feature is not only suppressed, but even reversed, as implemented in the Diffusion Model for Conflict Tasks (DMC) by Ulrich, Schröter, Leuthold, and Birngruber (2015). In this model, a diffusion process and an influence of the irrelevant stimulus feature are superimposed, with the cumulative influence of the irrelevant feature taking the form of a re-scaled Gamma density function which increases and declines during a single speeded decision. The decline of this function implies that the instantaneous influence of the irrelevant stimulus feature is reversed, that is, the first derivative of the function becomes negative (Ulrich et al., 2015; Figure B1 and eq. 5). Although the model is quite powerful in accounting for different shapes of delta plots, the assumption that in the course of a rapid decision an inhibitory instantaneous influence of an irrelevant stimulus feature is inverted to become facilitating (and vice versa) lacks plausibility in our view.

**Figure 1:**
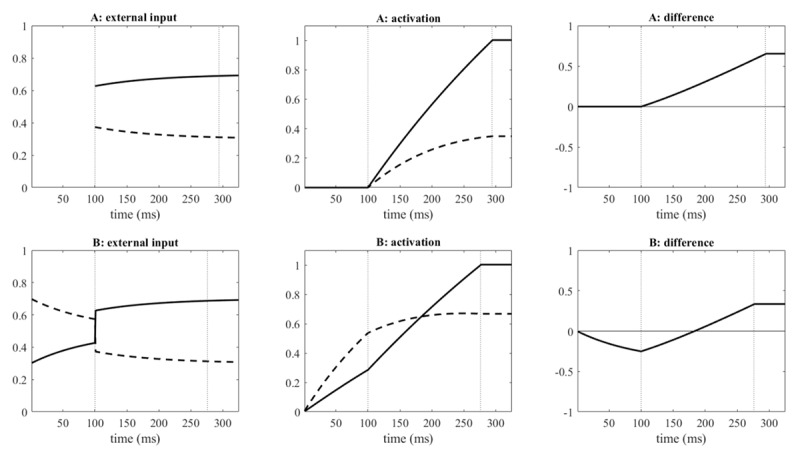
External inputs to response codes, response-code activations, and differences between response-code activations for Model A (upper row of graphs) and Model B (lower row of graphs). Continuous lines are for correct responses, dashed lines for error responses. The first vertical dotted line marks the end of the lead of the irrelevant input, the second vertical dotted line marks the end of the decision (reaching the threshold). Parameters were λ = .2, β = .2, σ_n_ = 0, I_rel_ = .7, ΔI_irr_ = –.2, δ = .1, *d* = –.1, *θ* = 1. The figures show an incongruent trial only.

Given suppression or passive decay of the instantaneous influence of an irrelevant stimulus feature, decreasing delta plots can also be explained without the assumption of an inversion of the irrelevant activation at longer reaction times. Specifically, they can result from a variable delay of processing the task-relevant stimulus feature during which the activation by the irrelevant stimulus feature is already partly suppressed (Miller & Schwarz, 2021; Wühr & Heuer, 2018). In fact, for the Simon task the assumption that processing of the irrelevant stimulus feature (stimulus position) starts earlier than processing of the relevant feature such as color or shape appears plausible, as argued, e.g., by Zorzi and Umiltá (1995).

The variety of shapes of delta plots does not only call for an explanation of decreasing delta plots which violate the typical relation between reaction-time means and variances, but also for an explanation of the very existence of different shapes. Such accounts are of basically two types, namely in terms of qualitative differences such as different processes or codes involved (e.g., De Jong et al., 1994; Gade, Paelecke, & Rey-Mermet, 2020; Wiegand & Wascher, 2005; Wühr & Biebl, 2011) or in terms of quantitative differences such as variations of temporal overlap of processing relevant and irrelevant stimulus features (e.g., Mackenzie, Mittelstädt, Ulrich, & Leuthold, 2022). Although these two claims can be contrasted (e.g., Töbel, Hübner, & Stürmer, 2014), qualitative differences such as the involvement of spatial codes in the horizontal but verbal codes in the vertical Simon task (Gade et al., 2020; Wühr & Biebl, 2011) will likely go along with quantitative differences of the temporal overlap of processing relevant and irrelevant features. Here we shall focus on the role of such quantitative variations for the shape of delta plots, that is, for the time course of congruency effects.

Variations of temporal overlap can result from faster or slower suppression (or decay) of the influence of the irrelevant stimulus feature or from staggered onsets of processing the relevant and irrelevant features. As noted above, for the classic Simon task processing of the irrelevant stimulus feature should lead processing of the relevant feature, but with a broader perspective on conflict tasks, processing delays of the different features should be variable and, depending on what these features are, the one or the other of them should lead systematically. Here we hypothesize that these variations give rise to a substantial part of the variety of delta plots observed for conflict tasks.

The most obvious test of this hypothesis requires the introduction of stimulus-onset asynchronies between relevant and irrelevant stimulus features. Previous studies have probed the effects of such experimentally induced delays for different conflict tasks. Independent of the details of the procedure, the typical result both for the Simon task and the Eriksen flanker task is a reduced increase or stronger decrease of the delta plot when the irrelevant feature is presented in advance of the relevant one (e.g., Burle et al., 2005; Hübner & Töbel, 2019; Mackenzie et al., 2022); with a sufficient lead of the irrelevant feature the congruency effect can even be absent beginning already at the shortest reaction times (Hübner & Mishra, 2013). In contrast, when the irrelevant feature is presented after the relevant one, delta plots increase with sometimes a delayed appearance of congruency effects (e.g., Burle et al., 2005; Mackenzie et al, 2022). An exception from this pattern has been found for the Stroop task, suggesting that other factors than temporal overlap – and delays of processing in particular – also modulate the shape of delta plots (Mackenzie et al., 2022).

Whereas stimulus-onset asynchronies (SOA) are a straightforward means to manipulate the order of processing onsets of relevant and irrelevant stimulus features, similar differences should also exist when irrelevant and relevant stimulus features are presented simultaneously, but processed differently before they start to exert an influence on response selection. For example, irrelevant stimulus position in a Simon task has a quite direct relation to the position of the left or right hand, whereas the relation of stimulus size to hand position is rather indirect. Thus, regarding the influence of the irrelevant stimulus feature on response selection, it appears plausible that the stimulus position leads the influence of a relevant feature such as color, whereas stimulus size lags. In a similar vein, Pratte (2021) speculated that the critical characteristic of those stimuli in the Eriksen flanker task that went along with decreasing instead of the typical increasing delta plots was their early processing in the visual cortex.

Previously we had added a variable lead of processing the irrelevant feature to a sequential-sampling model to account for decreasing delta plots as observed in the classic Simon task (Wühr & Heuer, 2018). Here we generalize this approach and make use of two extensions of a sequential-sampling model that allow not only a lead of processing the irrelevant feature of a conflict task, but also the relevant feature. The purpose of these models is to demonstrate the power of staggered onsets of processing different stimulus features as an account of the observed variety of delta plots.

## Leaky, competing accumulator models with staggered processing onsets

Sequential-sampling models vary in detail (cf. Bogacz, Brown, Moehlis, Holmes, & Cohen, 2006; Evans & Wagenmakers, 2020; Ratcliff, Smith, Brown, & McKoon, 2016; Smith & Ratcliff, 2004). A basic distinction is between models with a single state (or decision) variable, also designated as random-walk models, and models with two or more state variables, also known as accumulator models (cf. Heath, 1984; Smith & Ratcliff, 2004). Perhaps the most popular and mathematically elaborated sequential-sampling model is of the first type, the diffusion model originally proposed by Ratcliff (1978; cf. Ratcliff & Tuerlinckx, 2002; Voss, Nagler, & Lerche, 2013). Accumulator models are less popular; currently prominent among them is the leaky competing accumulator (LCA) model proposed by Usher and McClelland (2001). Here we use extensions of this model as we have done before (e.g. Wühr & Heuer, 2017, 2018, 2020, 2022) to explore the consequences of staggered onsets of processing relevant and irrelevant stimulus features for the shapes of delta plots. Although we focus on the LCA model, we want to stress that the principle of staggered onsets can be applied to other types of sequential-sampling models as well, where it should produce comparable results.

Our choice of the LCA model is motivated by the cumulative evidence of a tight relation between perceptual decisions and preparation of associated actions, as reviewed, e.g., by Verdonck, Loossens, and Philiastides (2021). For example, outcomes of perceptual decisions do not only depend on the sensory input, but also on the relative effort required by the associated actions (Hagura, Haggard, & Diedrichsen, 2017) and on the physical relation between them (Heuer, 1987; Wühr & Heuer, 2018). Furthermore, during rapid decisions, reflex gains are modulated depending on the forthcoming response (Selen, Shadlen, & Wolpert, 2012), and so is attention allocation to potential movement goals (Schonard, Heed, & Seegelke, 2022). In monkeys, decision-related increasing activity has been observed specifically in neurons of the lateral intraparietal cortex (LIP) whose response fields were related to the correct eye movement that served to indicate the direction of moving random dots (Steinemann, Stine, Trautmann, Zylberberg, Wolpert & Shadlen, 2022), and this decision-related activity was postponed when the eye-movement targets were presented with a delay (Shushruth, Zylberber, & Shadlen, 2022).

The notion of an intricate relation between perceptual decisions and associated actions gave rise to extensions of sequential-sampling models which relate muscle activity and overt movements to different thresholds (Servant, White, Montagnini, & Burle, 2015; Servant, Logan, Gajdos, & Evans, 2021) or which add movement preparation (Verdonck et al., 2021; Heuer, 1995) or overt movement (Lepora & Pezzulo, 2015) to the basic perceptual decision. Even without action-related extensions, however, accumulator models can be conceptualized in a way that respects the tight relation between perceptual analysis and motor preparation. Specifically, the formally defined accumulators of the models can be conceived as response codes that are progressively activated by the perceptual input. The activation of these codes reflects the current state of motor preparation, which includes the modulation of reflex gains (e.g., Brunia, Haagh, & Scheirs, 1985) and the direction of attention to potential targets (e.g., Baldauf & Deubel 2010; A.Heuer, Crawford, & Schubö, 2017). Different costs associated with the alternative responses in a choice task can be modelled in terms of different thresholds for the response codes (cf. Hagura et al., 2017), and the physical relation between alternative actions can be modelled in terms of forward or lateral inhibition between them (e.g., Heuer, 1987; Wühr & Heuer, 2018). Lateral inhibition between response codes as a free parameter is a distinguishing feature of the LCA model.

In the basic LCA model, noisy incremental activation is added to two response codes for correct and error responses, respectively, during each time step. A major component of the incremental activation is external input related to the task-relevant stimulus feature (‘relevant input’ for the sake of brevity). For conflict tasks, the basic model is extended by a second external input, which is related to the task-irrelevant feature (‘irrelevant input’). The irrelevant input differs from the relevant input by its dynamics: whereas the relevant input is time-invariant, the irrelevant input declines with the passage of time (cf. Hübner, Steinhauser, & Lehle, 2010; Kornblum et al., 1999; Ulrich et al., 2015; White, Ratcliff, & Starns, 2011; Zorzi & Umiltá, 1995), reflecting passive decay (e.g., Hommel, 1994) or active suppression (e.g., Van den Wildenberg et al., 2010). We previously added random delays of the relevant input to the model, which served to capture the declining delta plots observed in Simon tasks (Wühr & Heuer, 2018). Here we generalize the random delays of the relevant input to random temporal offsets between relevant and irrelevant external inputs, allowing not only lags of the relevant, but also of the irrelevant input. This generalization is essential for probing the hypothesis that different shapes of delta plots result from staggered onsets of processing relevant and irrelevant stimulus features.

We implemented staggered onsets of relevant and irrelevant inputs in two LCA models for conflict tasks, which differ only with respect to processing leading irrelevant input. Model A is similar to the model of Wühr and Heuer (2018) in that activation of response codes starts only when the relevant input becomes available. The irrelevant input declines during its lead, but has not yet an effect on the activation of response codes. In Model B, activation of response codes starts immediately when the first of the two inputs becomes available, similar to a network model proposed by Zorzi and Umiltá (1995).

The core of the LCA model is the difference equations that govern the incremental activations Δa_c_(i) and Δa_e_(i) of the two response codes for correct and error responses during each time interval i (cf. Usher & McClelland, 2001, Eq. 3):

1a \Delta {\rm{a_c}(i) = [I(i)- \lambda\ {a_c}(i) - \beta\ {a_e}(i)]\,\,(\Delta t/\tau ) + \xi (i)\,\,\surd \,(\Delta t/\tau )}

1b \Delta {\rm{a_e}(i) = [(1 - I(i)) - \lambda\ {a_e}(i) - \beta\ {a_c}(i)]\,\,(\Delta t/\tau ) + \xi (i)\,\,\surd \,(\Delta t/\tau )}

with self-inhibition gain λ, lateral-inhibition gain β, and Gaussian noise ξ(i) with zero mean and standard deviation σ_n_. I(i) and 1–I(i) are the external inputs which add to 1 (cf., Usher & McClelland, 2001, p.559); for I(i) = 0.5 they are thus identical for the correct- and error-response codes, and for I(i) > 1 the external input to the error-response code, 1–I(i), becomes negative. Thus, in addition to the lateral inhibition with gain β there can also be forward inhibition of the error-response code when the external input to the correct-response code becomes sufficiently strong.

The incremental activations of each response code (Eq. 1a, b) can be positive or negative. They are accumulated, beginning with a_c_(0) = a_e_(0) = 0. The total cumulated activation of each code is bounded to be non-negative:

2a {\rm{{a}_{c}}\left(i \right)=max\,\,\left[ \,0,\,\,{{a}_{c}}\left(i-1 \right)+\Delta {{a}_{c}}\left(i \right) \right]}

2b {\rm{{a}_{e}}\left(i \right)=max\,\,\left[ \,0,\,\,{{a}_{e}}\left(i-1 \right)+\Delta {{a}_{e}}\left(i \right) \right].}

A response is initiated when the activation of the respective response code, a_c_(i) for correct responses or a_e_(i) for errors, reaches a threshold *θ*. To cover the duration of sensory processing and motor outflow, a non-decision or residual time *R* with uniform distribution is added to the time needed for the decision. The assumption of a uniform distribution (with mean μ*_R_* and width w*_R_*) is somewhat arbitrary, but fairly common for sequential-sampling models (e.g. Ratcliff & McKoon, 2008), though not undebated (e.g. Verdonck & Tuerlinckx, 2016).

For all simulations we set Δt = 0.001 and τ = 0.1, so that Δt/τ = 0.01. In the simulations we computed the incremental activations and updated the response-code activations in two steps for each cycle i. In the first step only the external inputs I(i) and 1–I(i) and the noise ξ(i) were used and the activations of the response codes were updated preliminarily. In the second step, the preliminary updates of the activations of the response codes were used to apply self-inhibitions and lateral inhibitions to the incremental activations, and then the response-code activations of cycle i–1 were updated by these increments.

The basic LCA model is extended by the addition of a declining external input related to the task-irrelevant stimulus feature in conflict tasks and a variable offset *D* between processing the irrelevant and relevant inputs. These two extensions differ somewhat from those of the model of Wühr and Heuer (2018). First, rather than defining the total external input as a weighted mean of the time-invariant relevant and the exponentially declining irrelevant input, we here define the total input I(i) as the sum of two components: the time-invariant contribution I_rel_ of the relevant stimulus feature (relevant activation) and the additive time-varying contribution g(t)*ΔIirr of the irrelevant stimulus feature (irrelevant activation), with g(t) declining exponentially. More important than this difference to the previous model is the difference with respect to the variable offset *D*: rather than a uniform distribution between a maximal lead of the irrelevant feature and a zero lead, we now assume a uniform distribution with mean μ*_D_* and width w*_D_*. We define the time at which the relevant input becomes available as t = 0; with *d* < 0 the irrelevant input leads, and with *d* > 0 the relevant input leads. Models A and B differ with respect to the activation of the response codes for *d* < 0.

For Model A, the incremental activations are zero for t ≤ 0; activation of the response codes starts only when the relevant input becomes available. For t > 0 and *d* < 0, the external input is I(t) = I_rel_ + g(t) *ΔI_irr_, t = i*Δt, g(t)= *e*–(*t*–*d*)/δ with δ as the time constant of the exponential decline. In this model, the irrelevant input declines during the lead; at time t=0, when activation of the response codes starts, it is already smaller than its initial strength ΔI_irr_. With t > 0 and *d* > 0, that is, when the relevant input leads, I(t) = I_rel_ for t ≤ *d* and I(t) = I_rel_ + g(t)*ΔI_irr_, g(t) = e–(*t*–*d*)/δ, for t > d. In this case, the irrelevant input drives the incremental activations only after the (positive) delay *d*, beginning with its initial strength ΔI_irr_.

Model B differs from Model A for leading irrelevant input (*d* < 0), but not for leading relevant input (*d* ≥ 0), in that incremental activation of response codes starts already when the irrelevant input becomes available. For *d* < t < 0 (*d* < 0), external input to the incremental activation is I(t) = 0.5 + g(t) *ΔI_irr_ with g(t)= e–(*t*–*d*)/δ. At t = 0 the dummy input 0.5, which corresponds to an assumed relevant input that drives both response codes equally, is replaced by the actual I_rel_. Thus, during the lead of the irrelevant input, response codes are driven equally except for the additional influence of the irrelevant input. For an incongruent trial, Figure 1 illustrates the external inputs to the correct and error response codes, the total activations of these codes, and the difference between them.

As shown in Figure 1, in Model A the leading irrelevant input does not result in response-code activation, which starts only when the relevant input becomes available (after 100 ms in the example); however, during the lead time the irrelevant input declines. (The initial time course of the external input is not shown in Figure 1 because it is not yet an input to the response codes). In Model B, in contrast, response-code activation starts already when only the irrelevant input is available; as soon as the relevant input becomes available, there is a discontinuity in the signal because the dummy of 0.5 is replaced by the actual I_rel_. Even before the relevant input becomes available, the activation of both response codes increases, mimicking non-specific preparation. In addition, there is an asymmetry between the activations depending on the irrelevant input. Importantly, in incongruent trials there is an initially stronger activation of the error-response code so that the difference between the activations of the codes shows an initial “dip” (rightmost graph of the lower row). As noted by Zorzi and Umilta (1995), such a dip matches the well-established initial dip of the lateralized readiness potential in incongruent trials of a Simon task (cf. Leuthold, 2011, for a review).

**Figure 2:**
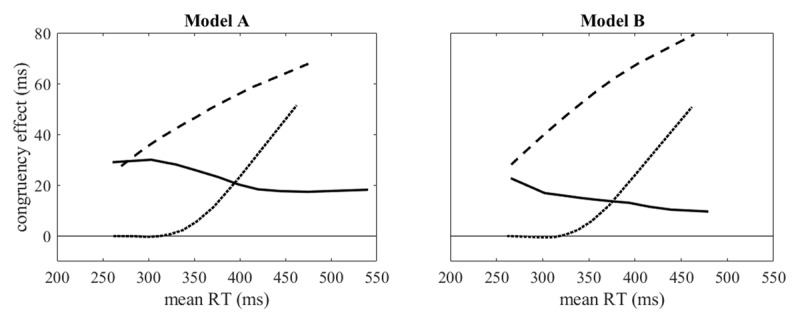
Different shapes of delta plots generated with Models A and B, using the parameters listed in Table 1, showing decreasing (continuous lines), increasing (dashed lines), and delayed (dotted lines) congruency effects. For each congruent (positive ΔI_irr_) and incongruent condition (negative ΔI_irr_) 100,000 trials were simulated.

Figure 2 illustrates basic types of delta plots that the models can produce. Each delta plot was derived from a congruent and an incongruent condition that differed only in the sign of ΔI_irr_, a simplification that we kept in fitting actual data. The main difference between the delta plots is in the mean temporal offsets μ*_D_*: with negative μ*_D_* (lead of the irrelevant input) declining delta plots can be observed, with μ*_D_* = 0 delta plots will generally be increasing, and with positive μ*_D_* congruency effects can be delayed. Note that both models are identical as long as the temporal offset *D* is always positive, which is the case for the dotted curves in Figure 2 (cf. Table 1). For Model A only the width of the distribution of the temporal offset was adjusted in addition to the mean to produce the declining delta plot. For Model B additional parameter adjustments were made to deal with the activation of the response codes during the lead of the irrelevant feature, namely an increase of the response threshold and an increase of I_rel_ to avoid an excessive number of errors, particularly in incongruent conditions. For the declining delta plot also the time constant of the decay of the irrelevant input was adjusted. The residual duration μ*_R_* was chosen as to make the shortest reaction times similar with the different model parameters. Thus, with Models A and B somewhat different parametrizations are required to produce basic shapes of delta plots.

**Table 1:** Parameters used to generate the delta plots of Figure 2.

	
	MODEL A			MODEL B		
	
PARAMETER	DECREASING	INCREASING	DELAYED	DECREASING	INCREASING	DELAYED

λ	.25	.25	.25	.25	.25	.25

β	.20	.20	.20	.20	.20	.20

σ_n_	.20	.20	.20	.20	.20	.20

I_rel_	.70	.70	.70	1.20	.70	.70

ΔI_irr_	±.15	±.15	±.15	±.15	±.15	±.15

δ	.10	.10	.10	.05	.10	.10

μ*_D_*	-.10	0	.20	-.30	0	.20

w*_D_*	.20	.10	.10	.20	.10	.10

θ	1.00	1.00	1.00	1.30	1.00	1.00

μ*_R_*	.10	.15	.15	.02	.15	.15

w*_R_*	0	0	0	0	0	0


Having shown that the models can produce different shapes of delta plots in principle, we applied them to data of four experiments with two different conflict tasks. In both tasks, the task-relevant feature was color. In one task, the task-irrelevant feature was stimulus size and in the other task, it was stimulus position (Simon task). Stimulus position has a quite direct relation to response position so that irrelevant input likely leads relevant input, resulting in the typical declining delta plots observed for the Simon task. In contrast, the relation of stimulus size to response position is rather indirect. It can be conceived as an instance of cross-modal correspondence although the associated features – size and position – are both visual (cf. Parise, 2016; Spence, 2011). Similar associations between magnitude of stimuli and lateral location of response keys have been observed for numbers in the well-known SNARC effect (Dehaene, Bossini, & Giroux, 1993) and for other stimuli that can be ordered on a magnitude dimension (e.g., Ren, Nicholls, Ma, & Chen, 2011; Walsh, 2003). Given the only indirect relation between stimulus size and response position, the irrelevant input could lag the relevant input, resulting in delayed congruency effects.

## Study 1: Size as task-irrelevant stimulus feature

We re-analyzed the data of two experiments that have been described in detail by Wühr and Seegelke (2018, Exp. 2) and Richter and Wühr (2022, Exp. 2). In the experiment of Wühr and Seegelke, the stimuli were green or red squares with side lengths of 20 or 40 mm; in the experiment of Richter and Wühr, the green or red squares had side lengths of 10, 20, 30, 40, 50 or 60 mm. Color was the task-relevant stimulus feature, and the mapping of the two colors to left-hand and right-hand responses was balanced across participants. In both experiments, the 4 or 12 color-size combinations were presented in random order in each block of trials (with the constraint of equal frequencies). Trials were classified as congruent when left-hand responses were made to small stimuli and right-hand responses to large stimuli; in incongruent trials, left-hand responses were made to large stimuli and right-hand responses to small stimuli.

For the present re-analyses, we collapsed responses with the left and right hand. Thus, in the experiment of Wühr and Seegelke (2018, Exp. 2) there were a congruent condition, in which the left hand responded to stimuli of 20 mm side length and the right hand to stimuli of 40 mm side length, and an incongruent condition, in which the left hand responded to 40-mm stimuli and the right hand to 20-mm stimuli. For the data of Richter and Wühr (2022, Exp. 2) we defined three pairs of congruent and incongruent conditions which differed with respect to the size difference. With the large difference, the congruent condition covered left-hand responses to 10-mm stimuli and right-hand responses to 60-mm stimuli and the incongruent condition left-hand responses to 60-mm stimuli and right-hand responses to 10-mm stimuli; with the intermediate size difference, the congruent and incongruent conditions included stimuli with side lengths of 20 and 50 mm, and with the small difference, stimulus sizes were 30 and 40 mm. For each participant and for each of these conditions, we computed the percentage of error responses, the mean and standard deviation of reaction times of correct responses, the bin means of the vincentized distributions of correct reaction times (with bins bounded by 20, 40, 60, and 80% of the distributions), and the delta plot for each pair of congruent and incongruent conditions. We dropped responses with RTs < 100 ms from all analyses reported in this article.

There are different ways of fitting sequential-sampling models to data, and none of them is without problems. Perhaps most important, for different types of sequential-sampling models and different fitting procedures, the parameters of simulated data sets cannot be reliably recovered (e.g. Hübner & Pelzer, 2020; Miletić et al., 2017; White et al., 2017). Among the other problems are the following: Different cost functions, which summarize the deviations between observed and predicted data, can produce different results because they place different weights on different aspects of the deviations. For locating the minimum of a multidimensional cost function, the risk of running into local minima is notorious. Even with a reasonable goodness-of-fit the parameter estimates can vary considerably (Miletić et al., 2017), suggesting “flat regions” of the multidimensional cost functions, that is, of the multidimensional surface where the minimum is sought. Fitting is particularly time-consuming for models for which there are no closed forms of the predicted distribution functions so that they have to be estimated by way of simulation of large numbers of trials. With such models, including the present ones, the search for a minimum of the cost function is analogous to finding the lowest point on a surface that changes its height at each measurement – with the variations being the stronger the smaller the simulated samples are. Thus, whatever fitting procedure is actually chosen, its results are afflicted with uncertainties.

Here we chose a rather pragmatic approach to fitting the models, similar to the one of Ulrich et al. (2015). We minimized the weighted sum of the squared deviations from the data we were primarily interested in, namely the sample means of the individual relative error frequencies and the sample means of the individual bin means. More specifically, the minimized cost function was

\begin{array}{*{20}{l}}
{C = 1000\sqrt {\frac{1}{{1.22m}}A}\ {\rm{with}}}\\
{A\; = \;\sum\nolimits_{j = 1}^m {\left[ {0.5{{\left( {{p_{ob.j}} - {p_{pr.j}}} \right)}^2} + {{\left( {P{{10}_{ob.j}} - P{{10}_{pr.j}}} \right)}^2} + {{\left( {P{{30}_{ob.j}} - P{{30}_{pr.j}}} \right)}^2}} \right.} }\\
{\qquad \left. { + {{\left( {P{{50}_{ob.j}} - P{{50}_{pr.j}}} \right)}^2} + {{\left( {P{{70}_{ob.j}} - P{{70}_{pr.j}}} \right)}^2} + 0.5{{\left( {P{{90}_{ob.j}} - P{{90}_{pr.j}}} \right)}^2}} \right]}
\end{array}

where *j* = 1, …,m are the experimental conditions, *p* is mean error probability, and *P*10, *P*30, *P*50, *P*70, and *P*90 are the sample means of the individual bin means in seconds (bins being bounded by the 2^nd^, 4^th^, 6^th^, and 8^th^ deciles of the individual distributions of reaction times of correct responses). Subscripts *ob* and *pr* indicate the observed and predicted data, respectively. Multiplication by 1000 improves readability. We started each search with 1000 simulated trials per condition, which were increased up to 100,000 trials. For the search of the minimal costs we used the MATLAB function *fminsearch*; the search was ended when a criterion was reached that included changes of the parameters and the function value (parameters of *fminsearch* were TolX = 0.3 and TolFun = 0.15). In some cases, we stopped a search also when successive runs with 75 or 100 iterations did not result in a reduction of costs. After the end of the search, we used the parameter estimates to re-compute the minimal costs and the predictions with another run of 100,000 simulated trials.

Table 2 presents the parameter estimates, which were constrained to be identical for congruent and incongruent conditions except for ΔI_irr_, which was positive for congruent and negative for incongruent conditions. Importantly, the mean temporal offset μ*_D_* was positive, indicating a lagged influence of the irrelevant stimulus feature on activation of response codes, and the width w*_D_* of the distribution of lags did not include 0. Thus, all temporal offsets *D* were positive, that is, in a range where Model A and Model B are identical. Therefore, the parameter estimates for both models differ only by chance. (The parameters of Model B are listed first because for the Simon task, described below, this model provided a better fit when temporal offset *D* could be negative.) Across both experiments, the parameter estimates were similar with few exceptions. These mainly related to the processing of the task-irrelevant feature. In the experiment of Richter and Wühr with six different stimulus sizes, the irrelevant signals were stronger initially than in the experiment of Wühr and Seegelke with only two different stimulus sizes (except for the smallest size difference), but declined more rapidly: according to the different time constants of the exponential decline, about 37% of its initial values was reached already after 31 rather than 71 ms. In addition, the width of the temporal-offset distribution was larger.

**Table 2:** Parameter estimates for Model B (in brackets for model A). Congruent and incongruent conditions differed only in the algebraic sign of ΔI_irr_, positive values for congruent and negative values for incongruent conditions, respectively. For the data of Richter and Wühr (2022) the absolute value of ΔI_irr_ could also vary across the three pairs of conditions with different size differences (sizes are italicized). Time parameters (δ, μ*_D_*, w*_D_*, μ*_R_*, and w*_R_*) are in seconds.


PARAMETER	WÜHR & SEEGELKE	RICHTER & WÜHR

λ (self-inhibition gain)	0.270 (0.269)	0.269 (0.269)

β (lateral-inhibition gain)	0.349 (0.345)	0.306 (0.305)

σ_n_ (standard deviation of noise)	0.282 (0.282)	0.272 (0.265)

I_rel_ (relevant input)	0.708 (0.707)	0.719 (0.719)

ΔI_irr_ (irrelevant input) *for sizes 20–40*:	± 0.064 (0.062)	

*10–60*:		± 0.127 (0.126)

*20–50*:		± 0.141 (0.141)

*30–40*:		± 0.042 (0.042)

δ (time constant for irrelevant input)	0.071 (0.069)	0.031 (0.031)

μ*_D_* (mean temporal offset)	0.180 (0.172)	0.208 (0.208)

w*_D_* (width of temporal-offset distribution)	0.053 (0.052)	0.093 (0.093)

θ (response threshold)	1.068 (1.070)	1.018 (1.018)

μ*_R_* (mean residual time)	0.180 (0.180)	0.176 (0.176)

w*_R_* (width of residual-time distribution)	0.059 (0.060)	0.063 (0.063)

costs	4.7 (4.7)	6.0 (6.0)


**Figure 3:**
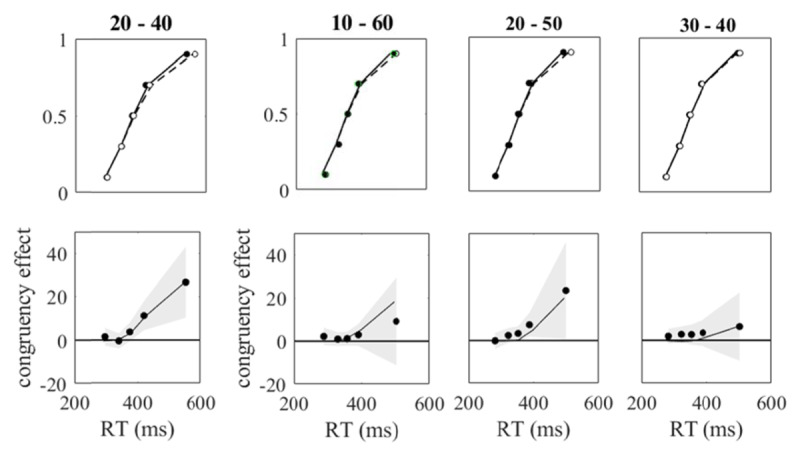
Upper row of graphs: observed (filled and open circles) and predicted (continuous and dashed lines) bin means in the format of cumulative distribution functions for congruent (filled and continuous) and incongruent (open and dashed) conditions. Lower row of graphs: Observed (filled circles) delta plots with 95% confidence range (t distributions) and predicted delta plots (continuous lines). Above each column of graphs stimulus sizes are given.

Table 3 shows the observed and predicted means of the individual error percentages (EP), of the individual mean reaction times of correct responses (M_RT_), and of the individual standard deviations of the reaction times (SD_RT_) for all pairs of congruent and incongruent conditions in both experiments. For the observed means, the standard errors are added. The predictions of Model A are in brackets; they differ from those of Model B slightly by chance. For Model B only one of the 24 predicted means deviated more than two standard errors from the observed mean, and six of the predicted means deviated more than one standard error. The observed and predicted distribution functions for reaction times as well as the delta plots are shown in Figure 3. The predictions are those of Model B, which were essentially indistinguishable from those of Model A. The observed and the predicted delta plots were quite similar. They gave evidence of delayed congruency effects, that is, there were no congruency effects for the shortest reaction times, but only for the higher quantiles of the reaction-time distributions.

**Table 3:** Observed and predicted mean error percentages (EP) as well as means of the individual means (M_RT_) and standard deviations (SD_RT_) of reaction times of correct trials (in milliseconds). For the observed means the standard errors are added. Predicted values of Model A are in brackets.

	
		OBSERVED		PREDICTED	
	
STIMULUS SIZES (MM)		CONGRUENT	INCONGRUENT	CONGRUENT	INCONGRUENT

20–40	EP (%)	2.3 ± 0.45	3.0 ± 0.55	2.1 (2.2)	3.0 (3.1)

	M_RT_ (ms)	394 ± 11.8	403 ± 12.1	392 (393)	400 (400)

	SD_RT_ (ms)	98 ± 9.7	107 ± 8.6	90 (91)	100 (100)

10–60	EP (%)	2.0 ± 0.39	3.0 ± 0.38	2.0 (1.8)	3.0 (2.3)

	M_RT_(ms)	374 ± 5.1	377 ± 5.4	368 (368)	377 (373)

	SD_RT_ (ms)	86 ± 5.3	88 ± 4.7	77 (76)	88 (83)

20–50	EP (%)	1.9 ± 0.28	2.2 ± 0.36	2.0 (1.6)	2.7 (2.3)

	M_RT_ (ms)	368 ± 5.1	375 ± 5.8	367 (368)	372 (373)

	SD_RT_ (ms)	86 ± 5.1	95 ± 5.9	77 (75)	84 (83)

30–40	EP (%)	2.1 ± 0.35	2.4 ± 0.42	2.1 (1.9)	2.4 (2.1)

	M_RT_ (ms)	370 ± 5.4	374 ± 5.8	369 (369)	370 (371)

	SD_RT_ (ms)	91 ± 4.8	90 ± 5.2	79 (78)	81 (80)


The re-analyses of the data of Wühr and Seegelke (2018, Exp.2) and Richter and Wühr (2022, Exp. 2) revealed delta plots for size as the irrelevant stimulus feature that showed no difference between congruent and incongruent conditions at the shortest reaction times. Rather a congruency effect emerged only at longer reaction times. This characteristic of the delta plots is consistent with the hypothesis of a lag of processing the task-irrelevant feature relative to processing the task-relevant feature, as indicated by the estimated mean temporal offset of the LCA models. A similar shape of delta plots has also been observed when irrelevant features were presented after the relevant ones in a Simon task or an Eriksen flanker task (Burle et al., 2005; Mackenzie et al., 2022). Our next step is fitting the models to a Simon task, for which we hypothesize a lead rather than a lag of processing the task-irrelevant stimulus feature when both features are presented simultaneously.

## Study 2: Position as task-irrelevant stimulus feature

In two Simon-task experiments, we varied the delay of the presentation of the task-irrelevant stimulus feature (position) relative to the presentation of the task-relevant feature (color). In Exp. 1, stimulus-onset asynchrony (SOA) was –200, –100, 0, +100, or +200 ms, and in Exp. 2, it was –300, –150, 0, +150 or +300 ms, with negative SOAs for leads of the irrelevant feature and positive SOAs for lags. By these SOAs, a constant external temporal offset between the relevant and the irrelevant stimulus feature was added to the variable internal one. As seems to be typical for the classical Simon task, the mean internal temporal offset should be negative rather than positive as found for size as the irrelevant stimulus feature. Therefore, differences between Model A and Model B should appear with SOA = 0, and even more so with negative SOAs. With positive SOAs, the total temporal offset will turn positive again, so that delta plots should increase above zero only at longer reaction times, like the delta plots seen with size as the task-irrelevant stimulus feature and in previous studies (e.g., Burle et al., 2005; Mackenzie et al, 2022).

### Methods

Twenty-nine students (20 female, 9 male) with mean age of 22 years (range 20 – 28 years) participated in Experiment 1, and 24 students (20 female, 4 male) with mean age of 24 years (range 20 – 32 years) participated in Experiment 2 for course credit. All participants were naïve with respect to the purpose of the study and reported normal (or corrected-to-normal) vision. One participant of each sample was excluded from the statistical analyses because of exceptionally long and variable reaction times; one of them had exceptionally large error rates as well so that the analyses were based on 28 and 23 participants, respectively.

Participants sat in a dimly lit room in front of a 17-inch monitor with unconstrained viewing distance of approximately 50 cm. A computer program written in ERTS language (Experimental Run Time System; BeriSoft, Frankfurt am Main, Germany) controlled stimulus presentation and response registration. Participants responded by pressing the left and right control keys of a standard keyboard with the index fingers of their hands held in parallel. The mapping of responses to the task-relevant stimulus feature – the red or green color of a square – was balanced across participants. The task-irrelevant feature was the left or right position of the square.

The two experiments were identical except for the SOAs of the relevant and the irrelevant stimulus feature. At the beginning of each experiment, the instructions were presented on the monitor. They described a typical sequence of events in each trial and the stimulus-response mapping. Participants read the instructions at leisure and pressed the space bar to start a practice block of 22 trials. The practice block was followed by ten experimental blocks, each consisting of 2 warm-up trials (which were not recorded) and 40 experimental trials (2 stimulus colors ⋅ 2 stimulus locations ⋅ 5 SOA conditions ⋅ 2 repetitions).

Each trial started with a blank screen for 500 ms, after which a gray square with a side length of 15 mm (approximately 1.4°) was presented at screen center. The square changed its color to red or green 500 ms after its onset and stayed for another 500 ms. Depending on the SOA condition, the square jumped to a lateral position located 80 mm to the left or right of fixation 200/300 ms before (SOA: –200/–300), 100/150 ms before (SOA: –100/–150), simultaneously with (SOA: 0), 100/150 ms after (SOA: +100/+150), or 200/300 ms (SOA: +200/+300) after its color changed from gray to green or red. Independent of the SOA, the square was shown (in two colors at two locations) for a total of 1,000 ms in each trial.

Response monitoring started with the presentation of the gray square at screen center and lasted for 2,000 ms. This allowed us to detect premature responses within a period of 500 ms before the square changed its color to red or green. An error message (in German) was presented for 2 seconds (a) if participants had pressed a wrong key, (b) if participants had responded prematurely or (c) if a response took longer than 1.5 s after presentation of the red or green color. Reaction time was measured from the onset of the task-relevant stimulus feature (green or red color of the square) or the onset of the task-irrelevant feature (lateral position of the square), whatever was first, until the keypress. In this way, we treat the experimentally defined temporal offset as if it were an internally generated offset, which differs from other studies in which presentation of the relevant feature triggered reaction-time measurements (e.g., Burle et al., 2005; Mackenzie et al., 2022). As the only consequence of this difference, reaction times for negative SOAs were longer than at SOAs ≥ 0 in the present experiments rather than shorter as when the SOA is not a part of the reaction time.

For the analyses we collapsed right-hand and left-hand responses so that there were 10 experimental conditions: for each of the 5 SOAs there were congruent (square and correct response key in the same relative position: left-left or right-right) and incongruent (square and correct key in different relative positions: left-right or right-left) trials. For each condition and participant, we computed the error rate and the mean and standard deviation of reaction times of correct responses.^1^ From the bin means of each pair of congruent and incongruent conditions, we computed the delta plot for each participant and SOA and characterized its overall increase or decrease across reaction-time bins by the slope of a linear regression (cf. Mackenzie et al., 2022). For these dependent variables, we report the results of statistical analyses.

Our main interest is in the variations of delta plots across SOAs and their modeling by way of staggered onsets of processing relevant and irrelevant stimulus features. Models A and B were fitted to the sample means of the relative frequencies of errors and of the bin means of each pair of congruent and incongruent conditions with a certain SOA by the same procedures as used for study 1. We fitted the models to the data of each SOA separately because fitting the models to all 10 conditions simultaneously turned out infeasible. Any attempt to keep the number of free parameters reasonably small requires the assumption that almost all model parameters are invariant across SOAs. This, however, is an unrealistic simplification because the different SOAs likely affect processing in more ways than just by variation of the temporal offset (cf. Mackenzie et al., 2022). For example, when the (task-irrelevant) position of the stimulus is presented in advance of the (task-relevant) color, it can serve as a warning signal for the forthcoming presentation of the relevant stimulus feature, and simultaneous presentation of stimulus features might change their processing as compared with successive presentation.

### Results

**Figure 4:**
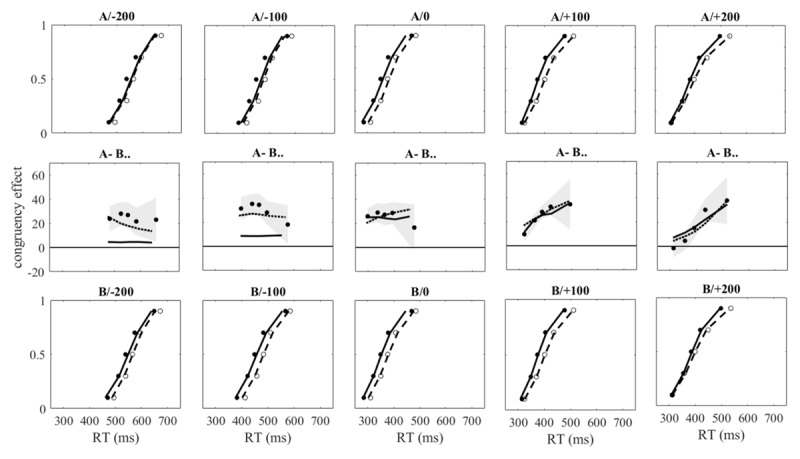
upper and lower row of graphs: Observed and predicted bin means of Exp. 1 plotted as cumulative distribution functions. Filled and open circles show observed means in congruent and incongruent conditions for each SOA, continuous and dashed lines show predicted means of model A (upper row) and Model B (lower row) for congruent and incongruent conditions, respectively. Middle row of graphs: Observed delta plots (filled circles) with 95% confidence band and predicted delta plots by Model A (continuous line) and model B (dotted line).

**Figure 5:**
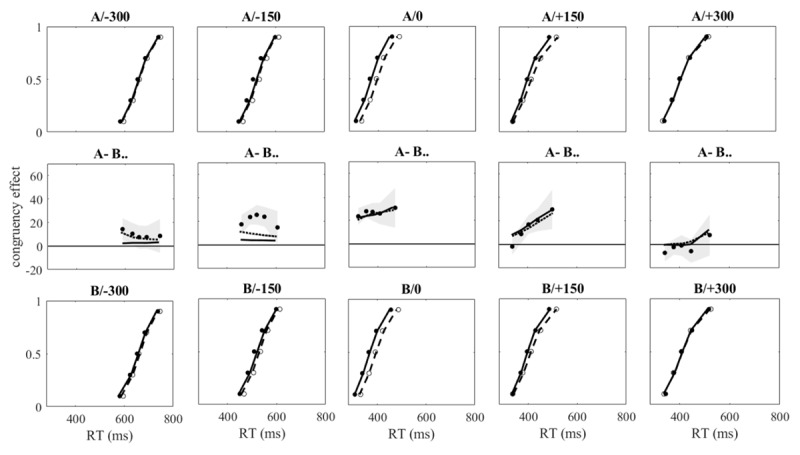
upper and lower row of graphs: Observed and predicted bin means of Exp. 2 plotted as cumulative distribution functions. Filled and open circles show observed means in congruent and incongruent conditions for each SOA, continuous and dashed lines show predicted means of model A (upper row) and Model B (lower row) for congruent and incongruent conditions, respectively. Middle row of graphs: Observed delta plots (filled circles) with 95% confidence band and predicted delta plots by Model A (continuous line) and model B (dotted line).

Tables 4 and 5 present the mean error percentages as well as the sample means of the individual means and standard deviations of reaction times of correct responses in Experiments 1 and 2. These three dependent variables were subjected to 2-way ANOVAs with the within-participant factors congruency and SOA. The sample means of the bin means are shown in Figures 4 and 5 as filled and open circles in the top and bottom row of graphs; the row of graphs in-between shows the delta plots derived from them. The slopes which served to indicate the overall increase or decrease of the delta plots were compared across SOAs by 1-way ANOVAs. When appropriate, we report the Greenhouse-Geisser-corrected *p* together with the correction factor ɛ; as effect size we report partial eta-squared.

**Table 4:** Observed and predicted results for Exp. 1; predicted results are those of Model B (predictions of Model A are in brackets).

	
		OBSERVED		PREDICTED	
	
SOA (MS)		CONGRUENT	INCONGRUENT	CONGRUENT	INCONGRUENT

–200	EP (%)	5.4 ± 0.91	9.5 ± 1.23	3.4 (5.3)	10.6 (9.4)

	M_RT_ (ms)	550 ± 5.5	575 ± 6.8	549 (557)	567 (561)

	SD_RT_ (ms)	71 ± 4.1	67 ± 3.7	63 (60)	59 (60)

–100	EP (%)	4.9 ± 0.77	11.2 ± 1.47	3.5 (4.0)	12.4 (11.2)

	M_RT_ (ms)	462 ± 6.2	492 ± 6.6	461 (469)	487 (478)

	SD_RT_ (ms)	74 ± 4.1	66 ± 3.6	64 (56)	64 (56)

0	EP (%)	1.3 ± 0.44	3.2 ± 0.79	0.7 (0.3)	3.7 (3.2)

	M_RT_ (ms)	361 ± 5.5	387 ± 6.2	358 (360)	385 (385)

	SD_RT_ (ms)	71 ± 3.7	71 ± 5.2	59 (50)	63 (61)

+100	EP (%)	0.8 ± 0.35	2.5 ± 0.50	0.7 (0.6)	2.6 (2.8)

	M_RT_ (ms)	384 ± 6.9	410 ± 7.2	383 (385)	409 (409)

	SD_RT_ (ms)	65 ± 4.9	72 ± 4.2	60 (60)	67 (66)

+200	EP (%)	1.3 ± 0.40	2.4 ± 0.50	1.0 (0.9)	2.8 (2.6)

	M_RT_ (ms)	395 ± 6.0	413 ± 8.7	394 (393)	410 (412)

	SD_RT_ (ms)	73 ± 4.0	87 ± 4.7	68 (71)	80 (81)


**Table 5:** Observed and predicted results for Exp. 2; predicted results are those of Model B (predictions of Model A are in brackets).

	
		OBSERVED		PREDICTED	
	
SOA (MS)		CONGRUENT	INCONGRUENT	CONGRUENT	INCONGRUENT

–300	EP (%)	7.1 ± 1.39	8.7 ± 1.38	5.9 (7.1)	8.9 (8.8)

	M_RT_ (ms)	656 ± 6.1	666 ± 7.3	656 (659)	663 (661)

	SD_RT_ (ms)	63 ± 3.1	70 ± 5.3	56 (53)	54 (54)

–150	EP (%)	6.3 ± 1.27	8.2 ± 1.15	4.9 (5.2)	9.3 (9.1)

	M_RT_ (ms)	518 ± 5.5	538 ± 7.3	521 (523)	530 (527)

	SD_RT_ (ms)	70 ± 5.2	63 ± 4.8	55 (51)	53 (50)

0	EP (%)	1.7 ± 0.43	6.1 ± 0.81	1.1 (0.5)	6.2 (6.3)

	M_RT_ (ms)	375 ± 6.8	403 ± 6.7	374 (373)	399 (398)

	SD_RT_ (ms)	64 ± 4.1	68 ± 4.1	51 (51)	54 (54)

+150	EP (%)	1.0 ± 0.26	2.0 ± 0.52	0.7 (0.5)	2.0 (1.8)

	M_RT_ (ms)	406 ± 7.9	422 ± 7.8	403 (402)	422 (420)

	SD_RT_ (ms)	67 ± 4.0	79 ± 5.7	57 (58)	79 (66)

+300	EP (%)	1.3 ± 0.35	1.0 ± 0.34	1.0 (1.1)	1.5 (1.8)

	M_RT_ (ms)	421 ± 10.2	421 ± 10.5	416 (417)	419 (419)

	SD_RT_ (ms)	75 ± 4.3	85 ± 4.6	63 (63)	67 (69)


Reaction times were slower in incongruent than in congruent conditions by 25 ms in Exp. 1, *F*(1,27) = 98.311, *p* < .001, \eta _{p}^{2} = 0.785, MSE = 441.133, and by 15 ms in Exp. 2, *F*(1,22) = 60.125, *p* < .001, \eta _{p}^{2} = 0.732, MSE = 213.836. Across SOAs, the congruency effect varied between 18 and 30 ms in Exp. 1, and between 0 and 27 ms in Exp. 2 with the more extreme SOAs. The interaction of congruency and SOAs was not significant in Exp. 1, *F*(4,108) = 1.424, *p* = .237, ɛ = .87, \eta _{p}^{2} = 0.050, MSE = 187.231, but in Exp. 2, *F*(4,88) = 7.553, *p* < .001, ɛ = .91, \eta _{p}^{2} = 0.256, MSE = 159.740. The shortest mean reaction time was observed with SOA=0 (374 and 389 ms in Exp. 1 and 2, respectively). It increased with increasing lead of the irrelevant feature (SOA < 0) by almost the duration of the lead, and it also increased with increasing lead of the relevant feature (SOA > 0). The main effect of SOA was significant in both experiments, *F*(4,108) = 921.347, *p* < .001, ɛ = .66, \eta _{p}^{2} = 0.972, MSE = 360.411, and *F*(4,88) = 878.423, *p* < .001, ɛ = .44, \eta _{p}^{2} = 0.976, MSE = 669.378, respectively.

Error percentages were larger in incongruent than in congruent conditions by 3.0 % in Exp. 1, *F*(1,27) = 26.525, *p* < .001, \eta _{p}^{2} = 0.496, MSE = 24.222, and by 1.7% in Exp. 2, *F*(1,22) = 10.846, *p* = .003, \eta _{p}^{2} = 0.330, MSE = 15.204. In Exp. 1 the congruency effect was stronger for SOA < 0 than for SOA ≥ 0, paralleling the difference in overall error rates. Both the main effect of SOA, *F*(4,108) = 40.805, *p* < .001, ɛ = .61, \eta _{p}^{2} = 0.602, MSE = 14.071, and the interaction of congruency and SOA, *F*(4,108) = 5.168, *p* = .003, ɛ = .70, \eta _{p}^{2} = 0.161, MSE = 12.412, were significant. In Exp. 2 the pattern was different, with the largest congruency effect at SOA=0 and slightly smaller effects at SOA < 0 despite larger overall error rates. For SOA = +150 ms the congruency effect was small and for SOA = +300 ms it was absent, as was the congruency effect for mean reaction time. Again both the main effect of SOA, *F*(4,88) = 24.937, *p* < .001, ɛ = .53, \eta _{p}^{2} = 0.531, MSE = 18.243, and the interaction of congruency and SOA, *F*(4,88) = 2.818, *p* = .049, ɛ = .71, \eta _{p}^{2} = 0.114, MSE = 11.902, were significant.

In Exp. 1, the mean standard deviation was smaller in incongruent than in congruent conditions for SOA < 0 and larger for SOA > 0; in Exp. 2 the smaller standard deviation in incongruent conditions was found only with SOA = –150 ms, but not with SOA = –300 ms. The interaction of congruency and SOA was significant for Exp. 1, *F*(4,108) = 3.589, *p* = .011, ɛ = .91, \eta _{p}^{2} = 0.117, MSE = 315.140, but not for Exp. 2, *F*(4,88) = 1.798, *p* = .148, ɛ = .85, \eta _{p}^{2} = 0.076, MSE = 372.919, whereas the main effect of congruency was significant only for Exp. 2, *F*(1,22) = 6.248, *p* = .020, \eta _{p}^{2} = 0.221, MSE = 219.899, but not for Exp. 1, *F*(1,27) = 0.663, *p* = .423, \eta _{p}^{2} = 0.024, MSE = 406.053 . In both experiments the main effect of SOA was significant primarily because of larger variability at SOAs of +150 ms and longer, *F*(4,108) = 3.361, *p* = .021, ɛ = .78, \eta _{p}^{2} = 0.111, MSE = 374.001, for Exp. 1 and *F*(4,88) = 5.383, *p* = .004, ɛ = .64, \eta _{p}^{2} = 0.197, MSE = 325.273, for Exp. 2.

The delta plots shown in the middle row of graphs of Figures 4 and 5 (filled circles) confirm previous observations (e.g., Burle et al., 2005; Mackenzie et al., 2022): When irrelevant features lead (SOA < 0), congruency effects tend to decrease as reaction times become longer (after an initial increase that is strongest at SOA of –150 ms in Exp. 2, weaker at SOAs of –100 and –200 ms in Exp. 1, and absent at SOA of –300 ms in Exp. 2). When relevant features lead (SOA > 0), congruency effects tend to increase at longer reaction times. The overall tendencies to decrease or increase are indicated by the slopes of the delta plots. For the SOAs from –200 to +200 ms in Exp. 1, the mean slopes (with SE) were –0.015 ± 0.046, –0.103 ± 0.054, –0.057 ± 0.049, 0.146 ± 0.058, and 0.193 ± 0.044, and their variation across SOAs was significant, *F*(4,108) = 6.507, *p* < .001, ɛ = .93, \eta _{p}^{2} = 0.194, MSE = 0.073. For Exp. 2 with a range of SOAs from -300 to +300 ms, the mean slopes were –0.013 ± 0.046, –0.019 ± 0.048, 0.045 ± 0.047, 0.198 ± 0.062, and 0.051 ± 0.047. Their variation across SOAs was significant as well, *F*(4,88) = 3.124, *p* = .026, ɛ = .84, \eta _{p}^{2} = 0.124, MSE = 0.056.

In addition to the observed data, Tables 4 and 5 present the predictions of both models; the predicted bin means are shown in Figures 4 and 5. As described in more detail below, the fit of Model B was better than that of Model A for SOA ≤ 0 (for SOA > 0, that is, for positive temporal offsets the models become formally identical as soon as the constant positive experimental temporal offset is larger than the largest negative internal offset). The predicted error rates and mean reaction times of Model B were close to the observed data, but the reaction-time standard deviations were consistently underestimated. A systematic underestimation was also observed when the models were fit to the data obtained with irrelevant stimulus size (cf. Table 2). The reasons for this underestimation are not obvious. Possibly, it results from differences between congruent and incongruent conditions that are not captured by different signs of ΔI_irr_, which were the only differences in fitting the models to these conditions. For example, the decrease of the influence of the irrelevant stimulus feature could be faster when it is incongruent with the response location than when it is congruent.

Tables 6 and 7 present the parameter estimates for Exp. 1 and 2. As indicated by the minimal costs, the fit of the models was better for positive SOAs than for negative ones. For negative SOAs (except for –300 in Exp. 2) the fit of Model B was better than that of Model A. For these SOAs, the predicted delta plots (middle row of graphs in Figures 4 and 5) of Model A deviated strongly from the observed ones (for the SOA of –150 ms, Exp. 2, the predicted delta plot of Model B did also deviate notably from the observed one). Therefore, we focus on the results obtained with Model B.

**Table 6:** Exp. 1, Parameter estimates for Model B (in brackets for model A). Congruent and incongruent conditions differed only in the algebraic sign of ΔI_irr_, positive values for congruent and negative values for incongruent conditions, respectively. Time parameters (δ, μ*_D_*, w*_D_*, μ*_R_*, and w*_R_*) are in seconds.

	
	SOA (MS)				

PARAMETER	–200	-100	0	+100	+200

λ	.256 (.259)	.241 (.273)	.263 (.253)	.207 (.232)	.237 (.235)

β	.211 (.226)	.220 (.232)	.194 (.234)	.210 (.215)	.243 (.232)

σ_n_	.184 (.199)	.202 (.203)	.222 (.234)	.257 (.266)	.235 (.258)

I_rel_	1.172 (.721)	.946 (.719)	.941 (.807)	.790 (.779)	.709 (.725)

ΔI_irr_	±.136 (.258)	±.151 (.242)	±.127 (±.221)	±.141 (±.185)	±.106 (±.094)

δ	.065 (.139)	.069 (.135)	.105 (.124)	.072 (.074)	.100 (.086)

μ*_D_*	–.104 (–.095)	–.084 (–.095)	–.081 (–.081)	–.101 (–.085)	-.059 (-.103)

w*_D_*	.170 (.131)	.194 (.121)	.240 (.132)	.215 (.227)	.203 (.219)

θ	1.271 (.447)	1.090 (.479)	1.074 (.736)	1.076 (.964)	1.011 (1.071)

μ*_R_*	.202 (.197)	.224 (.206)	.185 (.185)	.218 (.224)	.214 (.208)

w*_R_*	.076 (.140)	.090 (.124)	.081 (.107)	.093 (.049)	.119 (.103)

costs	9.6 (12.3)	8.5 (13.9)	7.5 (9.6)	3.0 (2.5)	4.0 (3.5)


**Table 7:** Exp. 2, Parameter estimates for Model B (in brackets for model A). Congruent and incongruent conditions differed only in the algebraic sign of ΔI_irr_, positive values for congruent and negative values for incongruent conditions, respectively. Time parameters (δ, μ*_D_*, w*_D_*, μ*_R_*, and w*_R_*) are in seconds.

	
	SOA (MS)				

PARAMETER	–300	–150	0	+150	+300

λ	.180 (.216)	.187 (.257)	.228 (.224)	.233 (.191)	.225 (.224)

β	.128 (.253)	.196 (.242)	.184 (.214)	.198 (.168)	.200 (.237)

σ_n_	.153 (.245)	.160 (.205)	.217 (.217)	.247 (.251)	.241 (.238)

I_rel_	1.215 (.726)	1.106 (.736)	.996 (.765)	.775 (.759)	.735 (.729)

ΔI_irr_	.054 (.263)	.055 (.277)	.146 (.256)	.099 (.104)	.064 (.207)

δ	.068 (.136)	.075 (.119)	.097 (.103)	.078 (.104)	.145 (.122)

μ*_D_*	–.120 (–.100)	–.110 (–.102)	–.098 (–.093)	–.096 (–.090)	-.082 (-.029)

w*_D_*	.136 (.110)	.143 (.108)	.209 (.081)	.307 (.266)	.229 (.163)

θ	1.584 (.596)	1.216 (.437)	1.040 (.687)	1.019 (1.072)	1.013 (1.008)

μ*_R_*	.198 (.172)	.221 (.211)	.209 (.190)	.241 (.231)	.244 (.242)

w*_R_*	.084 (.091)	.101 (.112)	.057 (.117)	.083 (.078)	.110 (.096)

costs	5.4 (4.1)	8.5 (10.0)	4.3 (6.3)	3.1 (3.5)	4.7 (4.0)


As the parameters of the present LCA models have functional meaning, their variations across experimental conditions should indicate functional variations. The caveat, however, is the noise of the parameter estimates and the risk of grossly wrong estimates, for example, when the cost minimization ended in a local minimum or somewhere on a flat multidimensional surface where the proper minimum could not be identified vis a vis noisy estimates of the surface. Despite these caveats, for some parameters there are systematic variations that are suggestive of functional variations.

First, the mean temporal offset μ*_D_*, which is an estimate of the internal component of the offset that is added to the experimentally controlled SOA, was negative for all conditions of both experiments, indicating an “internal” lead of the irrelevant feature. This is in contrast with the positive estimates for the experiments with size as irrelevant stimulus feature (cf. Table 1). For negative SOAs, when the irrelevant feature was presented before the relevant one, the lead tended to be larger. For the same conditions, the width of the distributions of the temporal offsets, w*_D_*, was smaller. This suggests that the relative lead of the irrelevant feature was longer and less variable when this feature was presented alone and before the relevant one than when it was presented while processing of the relevant feature was already on the way. For positive SOAs, the noise of the incremental activations was stronger than for negative SOAs, as indicated by a larger parameter σ_n_.

The relevant input I_rel_ declined for Model B, but not for Model A, as the SOA turned from negative to positive. Only for Model B the activation of response codes starts as soon as the irrelevant input becomes available (cf. Figure 1), that is, for conditions with SOA≤0. This only slightly asymmetric activation would increase the error rate, more so in incongruent than in congruent conditions. In fact, an overall higher error rate for negative than for positive SOAs is quite a consistent finding (Burle et al., 2005; Mackenzie et al., 2022). With Model B, there are two countermeasures to prevent too high error rates. The first is a strong relevant input I_rel_, which has the effect of not only activating the correct-response code, but also inhibiting the error-response code (forward inhibition). The second countermeasure is an increase of the response threshold *θ* for negative SOAs. (Note that with Model A the response threshold for the very same SOAs is reduced to take account of the increased error rate.)

The estimated initial strength of the irrelevant input, ΔI_irr_, behaved somewhat differently across the two experiments. In Exp. 2 it had a maximum at SOA = 0 and declined at negative and positive SOAs, that is, whenever the relevant and irrelevant stimulus feature were presented in succession rather than simultaneously. In Exp. 1, such a pattern was not present, but the variations of ΔI_irr_ across SOAs appeared more erratic. The time parameter for the decay of the irrelevant input, δ, showed the same variation in both experiments: decay was fast for negative SOAs, slow for SOA = 0, and faster again for positive SOAs except for the longest ones of +200 and +300 ms, where the decay became slow again.

## Discussion

Delta plots for conflict tasks show the time course of congruency effects across percentile bins of the reaction-time distributions. Their shape varies across different types of conflict tasks and across different variants of the same type of task. What causes this variety of time courses of congruency effects across different types and variants of conflict tasks? In the following, we discuss the validity of the hypothesis that guided the present analyses, namely that staggered onsets of processing task-relevant and task-irrelevant features can account for a major part of the diversity. In closing, we briefly touch upon some limitations of the models that we used for the formal representation of the hypothesis.

### Temporal overlap as a determinant of the shape of delta plots

Different shapes of delta plots could arise because of qualitative differences between conflict tasks such as different processes or different codes involved (e.g., Gade et al., 2020; Pratte, 2021; Wiegand & Wascher, 2005; Wühr & Biebl, 2011). However, different shapes of delta plots can also be observed when temporal offsets between presentations of relevant and irrelevant stimulus features are introduced for otherwise identical tasks, suggesting a critical role of temporal overlap (e.g., Burle et al., 2005; Hübner & Mishra, 2013; Hübner & Töbel, 2019; MacKenzie et al., 2022). These two perspectives on the shape variations of delta plots are not mutually exclusive because qualitative differences likely give rise to quantitative differences in the temporal overlap of processing relevant and irrelevant stimulus features. Vice versa, different time courses of congruency effects could reflect different mechanism (cf. Vallesi, Mapelli, Schiff, Amodio, & Umiltà, 2005). Overall, the role of temporal overlap for the time course of congruency effects can hardly be called into question.

Here we show a critical role of two manifestations of temporal overlap: the decay or suppression of the influence of the irrelevant feature and the temporal offset between the availabilities of relevant and irrelevant input for response selection. The declining influence of the irrelevant feature is widely accepted and an ingredient of several formal models (Hübner, Steinhauser, & Lehle, 2010; Ulrich et al., 2015; White, Ratcliff, & Starns, 2011; Zorzi & Umiltá, 1995). Critical evidence of such a decline has been the observation of decreasing congruency effects at longer reaction times. However, as explained in more detail above, increasing and decreasing influences of irrelevant stimulus features are not directly parallel to observed increases and declines of congruency effects. The reason is that the (theoretical) influence of irrelevant stimulus features represents instantaneous variations of the states of the processing system, whereas (observed) congruency effects are differences between reaction times, that is, between durations of processes that reflect variations of processing states in a cumulative manner. Therefore, a declining influence of irrelevant stimulus features cannot account for declining delta plots, except when the decline turns into a reversal of the instantaneous influence.

Declining delta plots can be modelled when a variable lead of processing the irrelevant stimulus feature is added to the assumption of its declining influence (Miller & Schwarz, 2021; Wühr & Heuer, 2018). Here we extend the notion of a variable lead of processing the irrelevant feature to a variable temporal offset between processing relevant and irrelevant features. Taking the LCA model as an example of a sequential-sampling model, we show that the appropriately extended model can produce different shapes of delta plots, including delta plots with a delayed congruency effect as we found with size as relevant stimulus feature or with a Simon task and a delayed presentation of the irrelevant stimulus position. Importantly, the models cannot only produce different shapes of delta plots as they can be found for different types of task and experimental conditions, but also mimic the observed distribution functions with a reasonable degree of accuracy.

### Staggered processing onsets in conflict tasks

Staggered processing onsets of relevant and irrelevant stimulus features have been induced experimentally in different ways, resulting in different shapes of delta plots. However, staggered processing onsets likely exist also when the stimulus features are presented simultaneously. For example, Zorzi and Umiltá (1995) argued for a faster processing of the irrelevant stimulus feature than of the relevant one in the Simon task. In fact, for the present models the negative estimates of the mean temporal offsets between relevant and irrelevant activations of response codes were consistent with this suggestion, as were previous estimates with a slightly different variant of an LCA model (Wühr & Heuer, 2018). In contrast, with stimulus size as the irrelevant feature the estimates of the mean temporal offsets were positive, suggesting a faster processing of the relevant stimulus feature. A conspicuous difference between position and size as irrelevant stimulus features appears to be the complexity of translating them into activations of responses with the left or right hand.

We are not aware of a systematic analysis of delta plots for different variants of conflict tasks from the perspective of hypothesized processing delays of task-relevant and task-irrelevant stimulus features for response selection – except for those studies in which temporal offsets are introduced explicitly. However, a few studies provide relevant data. For example, Mapelli, Rusconi, and Umiltá (2003) had their participants judge the parity of numbers 1–9 (without 5) which were presented to the left or right of the fixation point; responses were with the left or right hand. Thus, there were two irrelevant stimulus features, stimulus position and magnitude of the number presented. Both features could be congruent or incongruent with the response locations, allowing the determination both of the Simon effect (for stimulus position) and the SNARC effect (for number magnitude). Reaction-time distributions were split into 20% bins. The Simon effect varied significantly across bins and could even be reversed at the longest reaction times, whereas for the SNARC effect the variation across bins was not statistically significant (although the distribution functions for congruent and incongruent number magnitudes appear identical initially with an only slowly increasing SNARC effect as reaction times increase). The differences between Simon and SNARC effects have been confirmed by Gevers, Caessens, and Fias (2005), who also suggested that faster versus slower activations of spatial response codes could have played a role. This hypothesis is consistent with the present analysis according to which the differences between delta plots indicate a delay of processing the number magnitude and a lead of processing the number location relative to processing the relevant stimulus feature.

Recent findings by Pratte (2021) suggest that the relative complexity of mapping relevant and irrelevant stimulus features to response codes may not be the only factor that determines temporal offsets. Pratte studied Eriksen flanker tasks with different types of stimuli, but with targets and flankers always being of the same type. For color and motion stimuli, delta plots were increasing, but for oriented gratings and arrows they were decreasing (after an initial increase). In these experiments, the complexity of mapping relevant and irrelevant stimulus features on the left and right responses should be the same for each stimulus type, so there is no obvious reason to assume consistent temporal offsets. As noted by Pratte, decreasing delta plots were observed for those features that are processed early in the visual cortex (location and orientation), whereas increasing delta plots were found for features that require higher-level processing (color, motion; also letters as they have been used in several studies). Somewhat speculatively, low-level and rapid irrelevant spatial information could affect response selection earlier via the involuntary, automatic route of the dual-route model than the relevant spatial information via the intentional route.

The relation between experimentally controlled temporal offsets and different shapes of delta plots could be taken to suggest that shapes of delta plots could be used to infer temporal offsets that originate from different processing of simultaneously presented stimulus features. The findings of Pratte (2021), however, cast doubt on the validity of such inferences. First, there may be other and not yet known factors that shape delta plots. Second, and more important in the present context, temporal offsets exert their effects in combination with suppression of the irrelevant input. For example, without such suppression leads of the irrelevant input would not result in declining delta plots. Because of the combined effects of temporal offsets and suppression, the strength of suppression cannot be directly inferred from the slopes of delta plots (cf. Van den Wildenberg et al., 2010). For example, individual or group differences of the slopes could also reflect differences in temporal offsets rather than differences in the strength of suppression.

### The extended LCA model

We used extensions of the LCA model for a formal representation of the notion of staggered onsets of processing relevant and irrelevant stimulus features. Although we chose the LCA model for reasons explained in some detail above, we do not claim that it captures the existing data better than other models would do, particularly if staggered processing were added to them. Thus, it is not the model by itself that is important for our purpose, but what matters are the ideas that it formalizes – staggered onsets together with suppression (or decay) of irrelevant input.

Despite the specific purpose of the extensions of the LCA model and the reservations against the estimated parameters, the model-based analyses allow some tentative conclusions. Here we briefly discuss the differences between Models A and B, which differ only for leads of the irrelevant stimulus feature. For the corresponding data sets, Model B provided a somewhat better fit than Model A, though there was still room for improvement. This is most obvious with the SOA of -150 ms where the shape of the observed delta plot is not well captured by the model (see Figure 5), although LCA models with staggered onsets in principle can also produce delta plots with inverted U-shapes. According to Model B, response-code activation does not wait until the relevant input is available (as in the model of Wühr & Heuer, 2018), but starts as soon as the leading irrelevant input is available (as in the model of Zorzi & Umiltá, 1995). A preference for the assumptions implemented by Model B may not only be based on its better fit, but also on some other parallels to experimental findings. As noted by Zorzi and Umiltá (1995), the differences between response-code activations are consistent with the well-known early “dip” in the lateralized readiness potential observed in the incongruent condition of the horizontal Simon task. The early activation implemented in Model B also results in a higher error rate which is typical for conditions in which irrelevant stimulus features are presented before relevant ones (Burle et al., 2005; Hübner & Mishra, 2013; MacKenzie et al., 2022).

The models used for the present purpose have some limitations, and there are some obvious directions for improvement, which however, generally result in making them less tractable because of an increasing number of parameters. For example, the early activation implemented in Model B raises the error rate too much, and this excessive increase of the error rate is compensated by higher response thresholds and a stronger influence of the relevant stimulus feature, which has the effect of a forward inhibition of the error-response code. The excessive increase of the error rate could be avoided by downscaling of the external input during the time the relevant input is not yet available. An obvious limitation of the models, too, is that they cannot account for inversions of the Simon effect as reaction times increase. Different from, for example, the DMC of Ulrich et al. (2015), the instantaneous influence of the irrelevant stimulus can decline to zero in the present models, but not reverse. This could be remedied by the addition of another parameter, namely an asymptote for the decline.

## Data Accessibility Statements

The data used for Study 1 have been published on the Gesis/Datorium repository. The links to the data sets can be found in Wühr and Seegelke (2018) and in Wühr and Richter (2022). Both papers have been published with open access. The data used for Study 2 can be obtained from the third author (peter.wuehr@tu-dortmund.de) upon reasonable request.

The programs, which were used for performing model simulations, can be obtained from the first author upon reasonable request.

## Footnotes

There were 26 trials without response, and 11 trials with RT < 100 ms in the dataset from Experiment 1 (total number of trials = 11,600). There were 7 trials without response, and 5 trials with RT < 100 ms in the dataset of Experiment 2 (total number of trials = 9,600).
